# Evaluation of Immune Response and Protection Induced by V-ATPase Subunit F as DNA Vaccine Against *Leishmania tropica* (LCED Syrian 01) After Detection and Sequencing

**Published:** 2020

**Authors:** Amira Orabi, Mohammad Maarouf, Mustafa Alammori

**Affiliations:** Department of Biochemistry and Parasitology, Faculty of Pharmacy, Damascus University, Damascus, Syria

**Keywords:** BALB\c mice, DNA vaccine, *Leishmania tropica*, Parasite load, RT-PCR, V-ATPase subunit F

## Abstract

**Background:**

Leishmaniasis is one of the major emerging health problems worldwide and *Leishmania tropica* (*L. tropica*) is most prevalent in the Middle East due to conflict and environmental factors, and there is no effective prevention strategy available until now. An effective vaccine has not been developed to date. DNA vaccines are considered a promising approach to protect against this infection. In this study, since vacuolar (H+)-ATPase (V-ATPase) enzyme has an essential role in the life cycle of eukaryotes, V-ATPase subunit F gene has been chosen to design DNA vaccine and evaluate its immunogenicity in BALB\c mice.

**Methods:**

Genomic DNA was isolated from promastigote culture, synthesized complementary DNA (cDNA) after standardization of Polymerase Chain Reaction (PCR) conditions. The V-ATPase subunit F gene was placed into plasmid PCI. Then, recombinant plasmids were transformed into competent cells. Cloning was confirmed by PCR, restriction enzyme assays, and finally, DNA sequence analysis, after making miniprep from positive colonies and finally the gene was sequenced. BALB/c mice were immunized subcutaneously three times at an interval of two weeks with designed vaccine. BALB\c mice were challenged with 106 promastigotes of *L. tropica* 7 days post-immunization. IL-12, IFN-γ and IL-4 were quantified by RT-qPCR.

**Results:**

The present study proved the existence of subunit F gene in Syrian strain of *L. tropica* (LCED Syrian 01) promastigotes genome. Its expression was also proved in these parasites and the gene length was 414 *bp*.

**Conclusion:**

This study showed that vaccination of BALB\c mice with this gene induced partial protection against Leishmania by reduction of lesion size by 41.9% and parasite burden reduction by 3-log in the dLNs when compared with control group. IFN-γ\IL-4 was 1.6 after challenge test, so the immune response consisted of both Th1 and Th2.

## Introduction

The leishmaniasis is a group of diseases caused by protozoan parasites from more than 20 Leishmania species that are transmitted to humans by the bite of infected female phlebotomine sandflies (WHO, 2017)^1^. It is estimated that between 600 000 to 1 million new cases occur worldwide annually (WHO, 2018)^2^. Cutaneous Leishmaniasis (CL) is the most common form of leishmaniasis and causes skin lesions, mainly ulcers, on exposed parts of the body, leaving life-long scars. About 95% of CL cases occur in the Americas, the Mediterranean basin, the Middle East and Central Asia. In 2015, over two-thirds of new CL cases occurred in 6 countries of Afghanistan, Algeria, Brazil, Colombia, Iran and the Syrian Arab Republic. According to the WHO report in 2010, Syria is one of the countries affected most by cutaneous Leishmania caused by *Leishmania tropica* (*L. tropica*), with 25.000 new cases annually.

The Syrian Ministry of Health also published shocking statistics on the prevalence of this infection in Syria, where the number of new cases registered was 35000 cases in 2012 and 41000 cases in the first and second quarters of 2013 3. The deterioration of the health-care system in Syria along with the cessation of vector control programs and crowding created fertile ground for leishmaniasis outbreaks. These outbreaks proved to be international rather than localized. The unfortunate circumstances of refugee camps such as malnutrition, poor sanitation, and lack of health care crafted the perfect environment for leishmaniasis. The most reported species causing CL in Syria were *L. tropica* followed by *Leishmania major* (*L. major*) 4. Cutaneous lesions caused by *L. tropica* tend to be drier, have prolonged healing time and are more difficult to treat compared to lesions caused by *L. major* 5 regarding vector control to reduce transmission. Although a few therapeutic chemicals are now available, including antimonials, amphotericin-B, paromomycin and milte-fosine, some problems such as high toxicity, variable efficacy, inconvenient treatment schedules, costs and above all, drug resistance, remain to be addressed. Therefore, an efficient prophylactic vaccine is desperately needed in addition to new drug development 6.

Despite extensive efforts to develop an effective prophylactic vaccine, no promising vaccine is available yet. However, recent advancements in computational vaccinology on the one hand and genome sequencing approaches on the other hand generated new hopes in vaccine development 7. Vaccines development against this parasite is thus a major goal of international health agencies 8. DNA vaccination holds considerable promise for vaccination against different diseases in which Th1 responses and cell-mediated immunity are responsible for generating protection, for example against Leishmaniasis. Many antigenic molecules as vaccine candidates have been prepared and tested by several groups against Leishmania 9. Among vaccine types, the DNA vaccine seems to be the most promising one due to its ability to efficiently stimulate both humoral and cellular immunity in many infectious disease models 10. A DNA vaccine typically consists of a foreign gene, encoding a protein antigen of interest, cloned into a bacterial plasmid under the control of an appropriate promoter that can be injected into the skin or muscle of the host. After uptake of the plasmid, the protein is produced endogenously and intracellularly and the antigenic peptides can be presented on the cell surface in the context of both the MHC class I and MHC class II pathway 11.

In this study, V-ATPase subunit F was chosen, which is a part of the V-ATPase enzyme which has an essential role in the life cycle of the parasite. The eukaryotic V-ATPase is a 900 *kDa* membrane-intrinsic protein complex consisting of multiple subunits that functions as a rotary proton-pumping nano-motor 12. V-ATPase subunit F has a regulatory role and it is essential for coupling ATP synthesis with proton translocation across the membrane 13. In leishmaniasis, Th1-related cytokines production seems to be crucial for host control of parasite burden and clinical cure 14. IL12 secreted from DCs cells elicits Th1 cells to produce IFN-γ and IL-12 that are associated with cell-mediated immune response, including macrophage activation that kills the parasites by Nitric Oxide (NO) 15. IL-4 secreted from DCs cells elicits Th2 cells to produce different cytokines of IL-4 and IL-10 that inhibit the macrophages to kill the parasites; conversely, disease progression is generally associated with a T-helper-2 response 16. The predominance of these cell types determines the outcomes of infection 17.

V-ATPase subunit F has not been studied in *L. tropica* yet which is endemic in Syria. So, this study aimed to detect existence of V-ATPase subunit F gene in the genome of Syrian strain of *L. tropica*, to prove the expression of this gene in these parasites and to clone it into a eukaryotic expression plasmid for evaluating its efficacy against Syrian strain of *L. tropica* (LCED Syrian 01). BALB/c mice were immunized subcutaneously three times at an interval of two weeks with the designed vaccine. BALB\c mice were challenged with 106 promastigotes of *L. tropica* 7 days post-immunization. IL-12, IFN-γ and IL-4 were quantified by RT-qPCR.

## Materials and Methods

### Primer design for Polymerase Chain Reaction (PCR)

Because the sequence of V-ATPase subunit F in *L. tropica* is not known, an alignment for the sequences of the gene in other species was made using CLC free workbench 7 program. The procedure was done for *L. major* (Accession NO. XM_001681606.1), *Leishmania donovani* (*L. donovani*) (Accession NO. XM_003859-117.1), *Leishmania infantum* (*L. infantum*) (Accession NO. XM_003392244.1), and *Leishmania mexicana* (*L. mexicana*) (Accession NO. XM_003873108.1). The sequences are identical in terminal ends of the gene, so the common 18 bases from the beginning and 19 bases at the end of the gene were taken.

### Primers were designed using these applications

1- http://eu.idtdna.com/calc/analyzer: this application showed melting temperatures and the possibility of confirmation of self-dimers, hairpins, and heterodimers. The application determines the melting temperature of forwarding primer; it was 59.7°*C* and reverse primer was 60.9°*C.*

2- http://rna.lundberg.gu.se/cutter/: this application determines the restriction enzymes can cut the gene and our choice was XbaI and EcoRI. Restriction sites of either EcoRI or XbaI restriction enzymes were added on 5′ end of forward and reverse primers.

Final sequences for primers were:

Forward, 5′-AGAATTCATGCTGTCCCGTGCGCAG-'3
Reverse, 5′-TTTCTAGACTACTGCGAGTCCGAGGCG' 3

EcoRI and XbaI restriction sites included for direct cloning in the PCI mammalian expression vector are underlined which promotes constitutive expression of cloned DNA inserts in mammalian cells. https://www.promega.com//media/files/resources/protocols/techical-bulletins/. High purified primers were ordered from Alpha DNA, Montreal, Canada.

### DNA extraction

DNA was extracted from 5 *ml* of Leishmania *in vitro* culture (∼16×106 *cell/ml*). Promastigotes of a Syrian strain of *L. tropica* (Syrian 01) were supplied by LCEBS, Damascus, Syria. Promastigotes were cultured in a RPMI1640 medium (Lonza, Switzerland) and supplemented with 5% FCS (Fetal Calf Serum, Cytogen, GmbH, Germany). This culture was then incubated at 26°*C*. Genomic DNA was extracted from promastigotes by DNA extraction kit (Thermo scientific, Lithuania) according to the manufacturer’s instructions, and was separated by migration through agarose gel 1% detected by staining with ethidium bromide dye. Bands of DNA stained with this dye were visualized by illuminating the gel with UV light at one wavelength and recording at another. To determine DNA purity, measurement of absorbance was done by spectrophotometer. Absorbance readings were performed at 260 *nm* divided by the reading at 280 *nm*.

### Total RNA isolation and cDNA synthesis

Total RNA was extracted from harvested *L. tropica* promastigotes supplied by LCEB Syria. The extraction was carried out using TRI reagent (Sigma, St Louis, Mo, USA). RNA was quantified by NanoDrop- 1000 (Thermo Fisher Scientific MA, USA) according to the manufacturer’s instructions. Total RNA concentration was measured at 260 *nm* wavelength using spectrophotometer, and 500 *ng* was loaded into 1.5% agarose gel electrophoresis to inspect its integrity and quality after extraction. Total RNA (5 *μg*) was reverse transcribed into single-stranded cDNA using Revert Aid First Strand cDNA Synthesis kit (Thermo scientific, Lithuania) according to manufacturer's instructions. And the resulted cDNA (600 *ng*) was used as PCR template.

### Polymerase chain reaction (PCR)

DNA and cDNA of the V-ATPase subunit F gene were amplified by thermal cycler (Bio-Rad, USA) using PCR hot start green master mix (Thermoscientific, Lithuania) and the primers mentioned above. PCR protocol was optimized using gradient annealing temperatures (53–57°*C*). The reaction was performed in 25 *μL* of a mixture containing 1 *μL* of 10 *μM* from each primer, 2.5 *μL* of template DNA (175 *ng\μL*) or cDNA, 12.5 *μL* of PCR master mix and 8 *μL* of PCR water. Thermal cycling conditions were 95°*C* for 5 *min* followed by 35 cycles of 95°*C* for 1 *min*, annealing temperatures 53–57°*C* for 45 *s* and 72°*C* for 1 *min* and then 72°*C* for 5 *min* as a final extension. PCR products were loaded on 2% agarose gel, stained with ethidium bromide, and visualized on UV transilluminator.

### Ligation and transformation

PCR product containing V-ATPase subunit F coding sequence was purified using Gene Jet PCR Purification kit (Thermo scientific, Lithuania) according to manufacturer's instructions. Purified cDNA and the PCI mammalian expression vector (Promega, USA) were double digested with EcoRI plus XbaI. Ligation of the V-ATPase subunit F gene into the PCI plasmid was performed using T4 DNA ligase (Thermoscientific, Lithuania). Ligation reaction was prepared in 20 *μL* volume containing 5 *μL* of template (DNA extraction product), 1 *μL* of PCI plasmid, 2 *μL* of 10 x ligation buffer, 1 *μL* of T4 ligase and 11 *μL* of distilled water. This reaction was first incubated at 16°*C* overnight and then at 65°*C* in water bath for 5 *min* to stop the ligation reaction. Finally, the ligation product was stored at −20°*C*. The competent cells were prepared from the TOP10 strain of *Escherichia coli* (*E. coli*) bacteria using the calcium chloride method. *E. coli* 100 *μL* was cultured with 5 *ml* of new Luria-Bertani and then incubated at 37°*C* for 14 *hr*. The next day, 200 *μL* of these cells were cultured in 10 *ml* of LB broth and incubated at 37°*C* for three *hr*. After incubation, the culture was centrifuged at 7000 *rpm*, 4°*C* for 10 *min*. The supernatant was discharged and then 4 *ml* of cold calcium chloride (100 *mM*) was added to the pellet and mixed gently. The suspension was centrifuged at 7000 *rpm*, 4°*C* for 10 *min*.

The supernatant was discharged and 250 *μl* of cold calcium chloride was added to the pellet and mixed gently before being incubated on ice pieces for one *hr*. Following this, 10 *μL* of the ligation product was added to this suspension and incubated on ice pieces for 30 *min*. Then, the suspension for heat shock was incubated at 42°*C* for 90 *s* and then immediately transferred onto ice. Subsequently, 500 *μl* of LB broth, without any antibiotics, was added and incubated at 37°*C* for one hour. These cells were cultured on plates of Luria-Bertani (LB) agar medium containing 100 *mg/ml* of ampicillin. The plates were incubated at 37°*C* for 16 *hr*. Transformation products were amplified by PCR and electrophoresis of the products on 2% agarose gel to investigate the positive colonies. Recombinant plasmids (PCI- V-ATPase subunit F) were extracted from positive colonies using GF-1 plasmid extraction kit (Vivantis, Malaysia) according to manufacturer's instructions. The recombinant plasmid (PCI-subunit F) was digested by EcoRI and XbaI enzymes. The double digestion reactions were prepared in 30 *μL* volumes containing 4 *μL* of the plasmid extraction product, 9 *μL* of water, 4 *μL* of tango buffer (Thermo Scientific, Lithuania), 1 *μL* of EcoRI and 2 *μL* of XbaI enzymes. These reactions were incubated at 37°*C* for 16 *hr*. The products of digestion were analyzed by electrophoresis on 2% agarose gel to ensure that the cloning is positive.

### Sequence analysis

Full-length double-stranded sequence analysis was performed on purified recombinant plasmid PCI-sub-unit F on automated sequencer (ABI PRISM BigDye Terminator Cycle Sequencing Ready Reaction Kit, Perkin-Elmer, Foster City, USA).

### Experimental section

***Mice:*** Female BALB\c mice were one month-old at the onset of the experiments. Mice were purchased from the Scientific Research Center-Damascus-Syria. During experimentation, mice were kept under conventional conditions in an isolation facility. Mice were divided into 2 groups, each one contained 25 mice.

### Mice vaccination

BALB/c mice were immunized three times at 2-week intervals by the administration of 100 *μg* of V-ATPase subunit F vaccine suspended in 100 *μL* of Phosphate Saline Buffer (PBS) subcutaneously (s.c.). As a control, 25 mice were inoculated with empty plasmid (CO-PLA).

### Parasite challenge

Parasite challenge was done by subcutaneous inoculation in the left foot-pad with 106 stationary-phase promastigotes of *L. tropica* (LCED Syrian 01) 7 days post vaccination. Footpad thicknesses was measured with a metric caliper (Mitutoya, Kawasaki Kanagwa, Japan), and calculated as thickness of the left footpad minus thickness of the right footpad. Comparison was done between the vaccinated mice group and the control group.

### Estimation of parasitic load

The parasite load was determined by limiting dilution assay method. The number of viable parasites was determined from the highest dilution at which promastigotes could be grown up to 7-day incubation at 26°*C*. Parasite load was determined in the site of inoculation and in dLNs of BALB\c mice. Dermis of the inoculated foot-pad and dLNs tissues were homogenized separately in 500 *μl* of RPMI 1640. In this study, 24 well cell culture plates were used. Each homogenate was diluted by 10-fold serial dilutions in consecutive wells. After 7 days of incubation at 26°*C* with CO_2_ 5%, the wells were examined for motile promastigotes using an inverted microscope.

### Real-time RT-PCR

IL-12, IFN-γ and IL-4 were quantified by q-PCR 2,4 and 6-week post-infection; 3 mice were killed at each time point for monitoring the cytokines gene expression in dLNS, using a quantitative PCR assay. Lymph nodes were removed by using different scissors or scalpels to avoid contamination and were minced with Potter grinders and then carefully homogenized in 1.5 *ml* microtubes and filtered. RNA was extracted from dLNs of each mice group using a Gene JET RNA purification extraction kit. A trace of genomic DNA was removed with an RNase free-DNAase set. RNA (2 *μg*) was reverse transcribed using reverse transcriptase (200U). Subsequent real-time PCR was performed on Step One real-time PCR system (Applied Biosystems) using SYBR GREEN (sybr-green master mix), and 20 *ng* of cDNA as a template (cDNA Omniscript RT kit; Qiagen). The following are the forward and reverse primers:

IFN-γ-RV ′5-TGGCTCTGCAGGATTTTCATG-3′
IFN-γ-FW ′5-TCAAGTGGCATAGATGTGGAAGA A-3′
IL-4-RV ′5-GAAGCCCTACAGACGAGCAGCTCA-3′
IL-4-FW ′5-ACAGGAGAAGGGACGCCAT-3′
IL-12 -RV ′5-AACTTGAGGGAGAAGTAGGAATGG-3′
IL-12-FW ′5-GGAAGCACGGCAGCAGAATA-3′
HPRT-RV ′5-CCAGCAAGCTTGCAACCTTAACC A-3′
HPRT-FW ′5-GTAATGATCAGTCAACGGGGGAC-3′

The mRNA expression levels were normalized to the hypo-xanthine phosphor ribosyltransferase (HPRT) gene and calculated as the n-fold difference of the expression in activated cells compared with its naïve counterpart. Estimation of relative expression of the respective mRNA was made by RT-PCR and primers of IL-12, IL-4, IFN-γ. PCR was carried out in a final concentration of 10 *μm* forward and reverse primers, 1×SYBR GREEN reaction master mix (Applied Biosystems, USA), and 5 *μL* of template DNA 110 *ng\μL*. PCR conditions were as follows: an initial denaturation step at 95°*C* for 2 *min* followed by 35 cycles of denaturation at 95°*C* for 1 *min* and annealing/extension at 61°*C* for 1 *min*. Reactions were processed and analyzed with an Mx3005P (Agilent Technologies, USA). Gene expression was calculated by relative quantitation using the comparative Ct method (ΔΔCt), as described^17^, with threshold set at 0.02. Gene expression was expressed as fold change (2-ΔΔCt), in relation to samples from control group, used as calibrators.

### Statistical analysis

The values expressed by average X and SD were compared by the mixed ANOVA. The results were considered statistically significant when p<0.05

## Results

### Evaluation of DNA and RNA quality and purity

**Figure 1.**
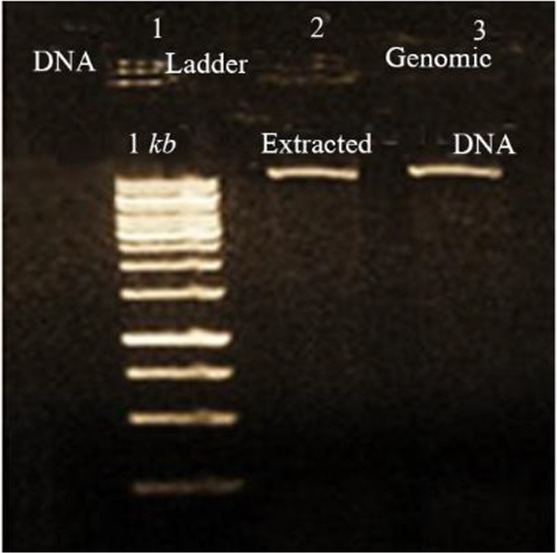
Electrophoresis of extracted *L. tropica* DNA, lane 1: DNA 1 *kb*, lane 2, 3: extracted *L. tropica* DNA.

**Figure 2.**
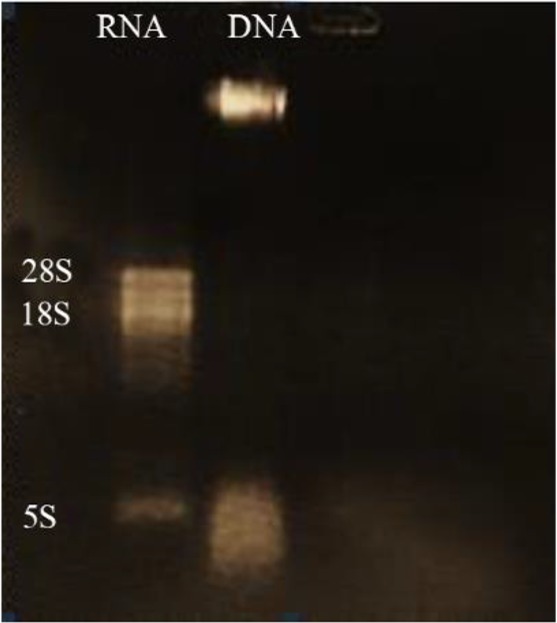
Electrophoresis of extracted *L. tropica* RNA, lane 1: *L. tropica* RNA, lane 2: extracted *L. tropica* DNA.

Extracted DNA electrophoresis on a 1% agarose gel showed only one band (Figure 1). The purity of the DNA was 1.8. Extracted RNA electrophoresis on 1.5% agarose gel showed a standard profile and there was no contamination with genomic DNA (Figure 2). The total RNA 260/280 ratio was 2.

### Polymerase chain reaction

**Figure 3.**
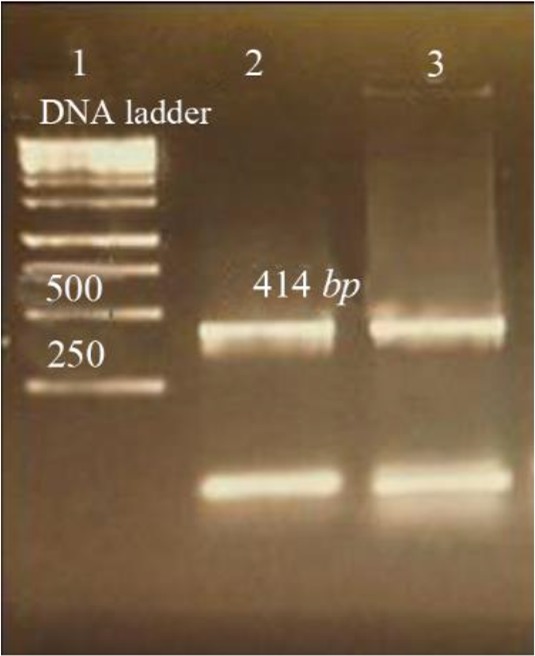
Amplification products of *L. tropica* V-ATPase subunit F on 2% agarose gel electrophoresis stained with ethidium bromide. Lane 1: DNA ladder 1 *kb*, lane 2: V-ATPase subunit F amplification product, lane 3: V-ATPase subunit F- cDNA amplification product.

PCR protocol was optimized using gradient annealing temperatures and the most suitable annealing temperature was selected which was 54.5°*C*. Gel electrophoresis of PCR products on the level of DNA and cDNA showed only one band and its size was approximately 414 base pair (Figure 3).

### Cloning and sequencing of the V-ATPase subunit F cDNA

**Figure 4.**
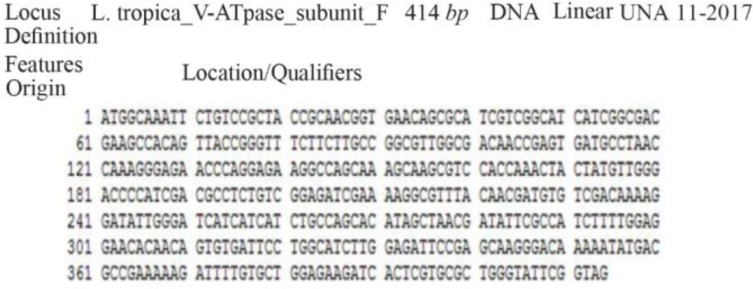
Amplification products of transformation PCR products on 2% agarose gel electrophoresis stained with ethidium bromide. Lane 1: DNA ladder 1 *kb*, lane 2, 3: V-ATPase subunit F positive amplification product.

**Figure 5.**
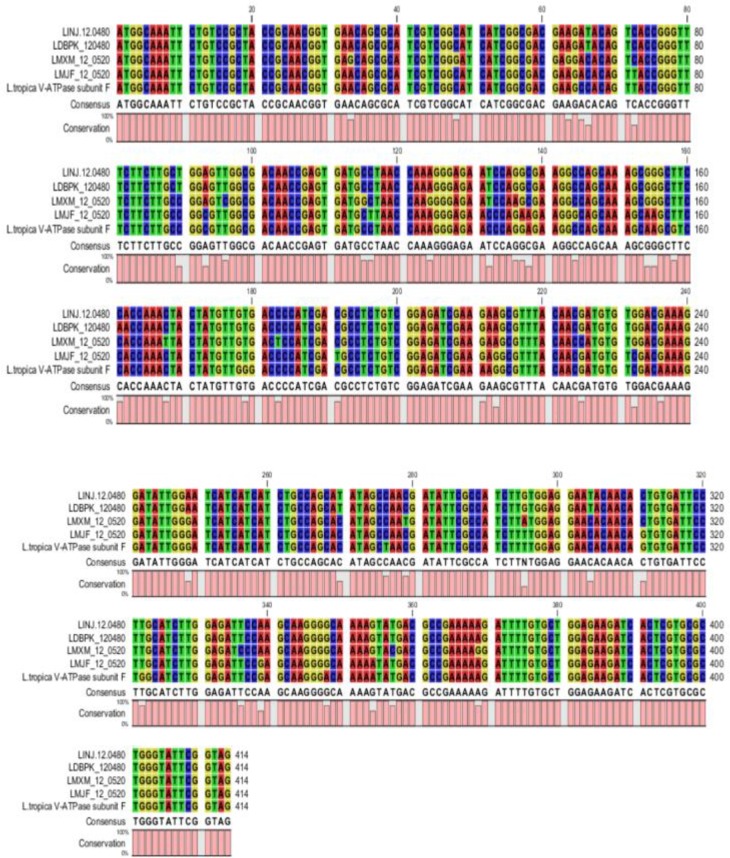
Amplification products of extracted plasmid on 2% agarose gel electrophoresis stained with ethidium bromide. Lane 1: DNA ladder 1 *kb*, lane 2: undigested plasmid band, lane 3,4: digested plasmid shows a band of V-ATPase subunit F gene and a band of the plasmid.

**Figure 6.**
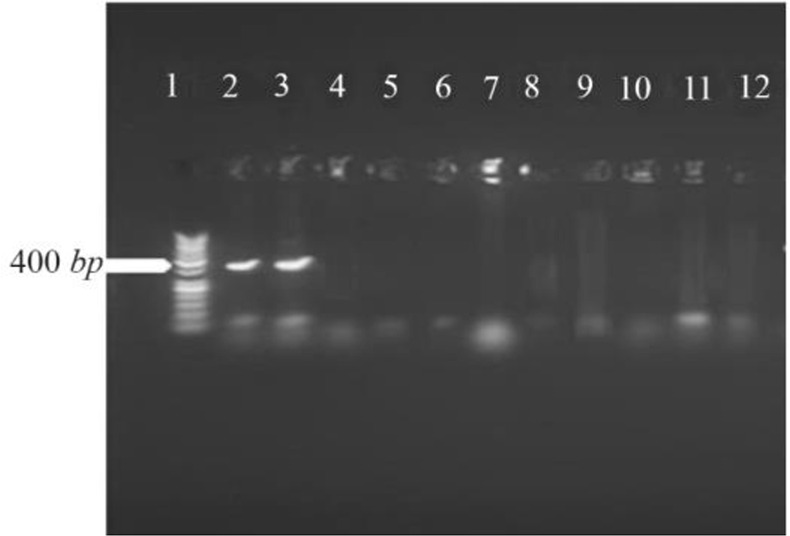
Sequencing of V-ATPase subunit-F gene in *L.tropica.*

**Figure 7.**
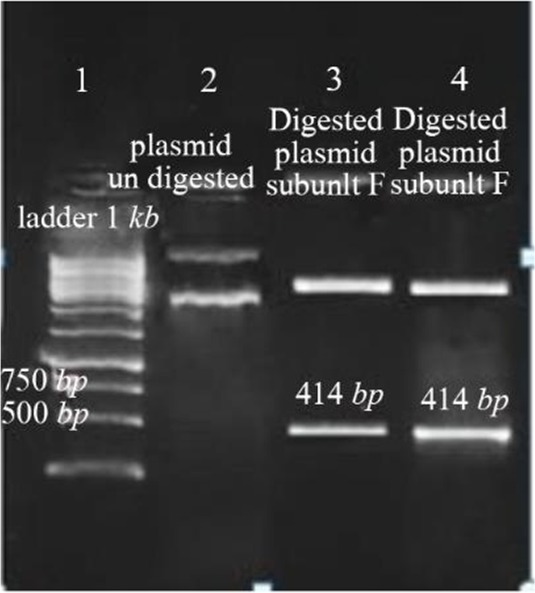
Nucleotide sequence of *L. tropica* V-ATPase subunit F and sequence in other *Leishmania* species, *L. infantum* (*LINJ*), *L. major* (*LMJF*)*, L. donovani* (*LDBPK*), *L. mexicana* (*LMXM*).

The results of PCR products of 12 colonies have shown that two of them were positive and showed the band of subunit F gene (Figure 4). Plasmids were extracted from positive colonies, and the electrophoresis of the PCR product showed two bands for plasmid and V-ATPase subunit F gene (Figure 5). In an effort to clone and characterize the *L. tropica* V- ATPase subunit F gene, a cDNA clone was identified and sequenced. The sequence was submitted to the Genebank under accession number MH124206.1. Figure 6 shows the complete nucleotide sequence of the 414 *bp* cDNA insert and the deduced amino acid sequence predicts a protein containing 138 amino acids and a predicted molecular mass of 128.22 *kDa*. The V-ATPase subunit F which predicted amino acid sequence was compared with the sequence of proteins coded by cDNA of V-ATPase subunit F in other species of Leishmania such as *L. infantum*, *L. major*, *L. donovani*, *L. mexicana* using vector NTI program. Interestingly, *L. tropica* V-ATPase subunit F shows significant similarity with V-ATPase subunit F of *L. infantum* (94%), *L. major* (93%), *L. donovani* (94%) and *L. mexicana* (92%) (Figure 7).

### The effect of mice vaccination on infection development and parasite load

Footpads swelling in vaccinated mice were compared with that of control; footpads thickness was measured and parasite load analysis was done in the inoculation site. A decrease in footpads swelling was observed by 41.90% in vaccinated group compared with control group after 6 weeks from the last vaccination. The parasite load was 107 in the inoculation site of vaccinated group and 108 in the draining lymph nodes.

### IFN-γ, IL-12, IL-4 gene expression analysis by RT-qPCR

In order to determine whether the cellular immune response induced by vaccination is associated with a cytokine expression, the gene expression of interleukin-12 (IL-12), interferon gamma (IFN-γ) and interleukin-4 (IL-4) was assessed in the dLNs of each vaccinated group 2,4 and 6 weeks after the last dose of vaccination. qPCR showed a significant increase in the expression of all immunological markers; about 9 fold increase (p=0.001) in IL-12, about 8 fold increase (p=0.002) in IFN-γ and about 5 fold increase (p=0.01) in IL-4 was observed comparing to control group.

## Discussion

V-ATPase subunit F is a regulatory subunit in the vacuolar (H+)-ATPase enzyme and it exhibits two conformations, a “retracted” form in the absence and an “extended” form in the presence of ATP 18. According to the important role of subunit F in V-ATPase in Leishmania and other eukaryotes, the current research aimed to detect the presence of V-ATPase subunit F in the *L. tropica* genome which is endemic in Syria. An alignment has been made for the sequences of the gene in other Leishmania species to design primers and optimize the conditions of the PCR reaction to detect the presence of the gene in (LCED Syrian 01) strain of *L. tropica*. The annealing temperature was 54.5°*C* for the designed primers. Gel electrophoresis of genomic DNA proved that there is no degradation in the extracted DNA and the absorbance showed high purity of genomic DNA and RNA (Good-quality DNA will have an A260/A280 ratio of 1.7–2.0 and pure (total) RNA has a ratio of 1.9–2.0). Gel electrophoresis of PCR product on the level of DNA and cDNA showed only one band; its size was approximately 414 base pair which was identical to the V-ATPase subunit F in other Leishmania species so the primers are specific for this gene and the sequencing proved that the gene length was 414 base pair. Due to these results, the present study proved that V-ATPase subunit F is a part of *L. tropica* genome and it has an expression in the parasite and also the sequencing of the gene showed a high percentage of similarity with V-ATPase subunit F of *L. infantum* 94%, *L. major* 93%, *L. donovani* 94% and *L. mexicana* 92%. This research hasn't been compared with other researches since there are no studies on this gene in other species of Leishmania. This strong similarity increases its potential as a vaccine for more than one type of leishmaniasis.

**Figure 8.**
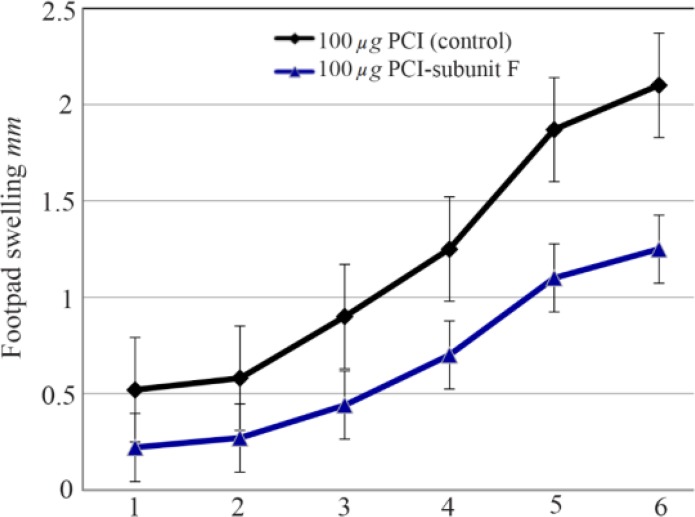
Footpad swelling in BALB/c mice immunized SC, 3 times in 2 weeks intervals, with empty plasmid as control, V-ATPase subunit F DNA vaccine, after challenge with virulent *L. tropica* promastigotes. The mice were challenged in the left footpad with 10^6^ *L. tropica* promastigotes, 2 weeks after the last booster. The footpad thickness of mice was then measured on both footpads for 6 weeks. Each point represents the average increase in footpad thickness. p<0.05 indicates that the values of marked immunized mice are significantly different from those received empty plasmid.

**Figure 9.**
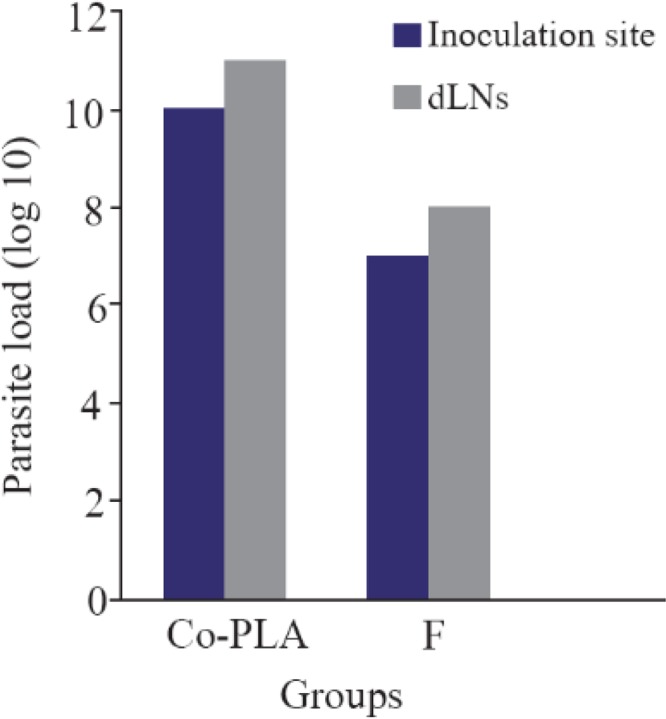
Monitoring of the parasitic load in the site of inoculation and in dLNs in BALB/c mice after intradermal inoculation of 10^6^ *L. tropica* promastigotes into the left foot dermis. Parasite burdens were analyzed at week 6 after the last dose of the vaccination by limiting dilution in the lymph node and left foot pad.

**Figure 10.**
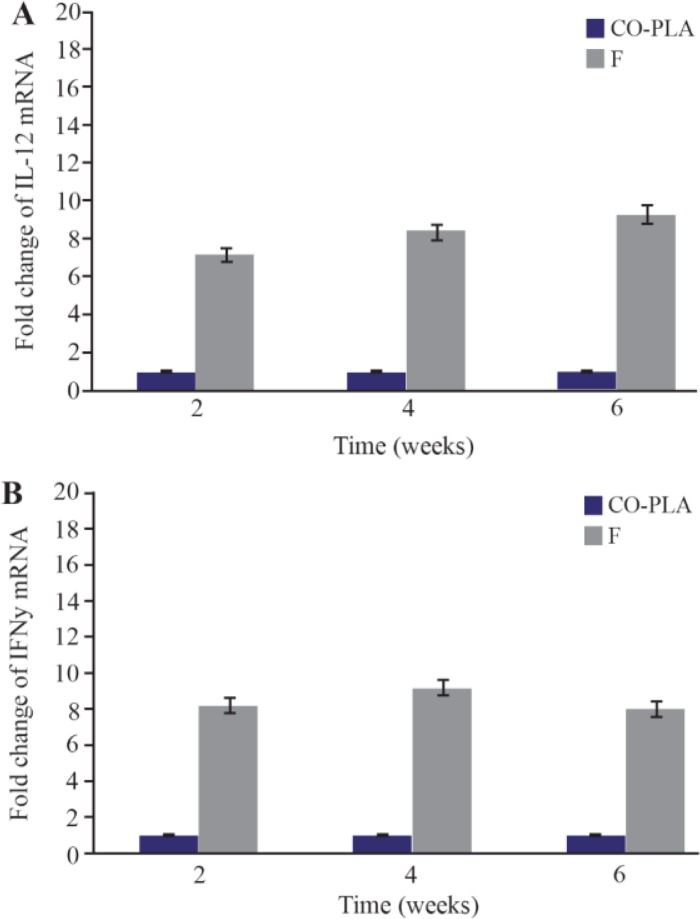
IL-12 (fig. 10-A), IL-4 (fig. 10-B), IFN-γ (fig. 10-C) expression in the draining lymph nodes obtained from BALB\c mice inoculated with 100 *μg* V-ATPase subunit F DNA vaccine. The messenger RNA (mRNA) for *IL-12, IL-4, IFN-γ* genes were determined by RT-PCR. As geometric mean±SD (three dLNs). The relative quantification was performed by the comparative Ct method (△△Ct), using lymph node from uninfected animals, respectively, as calibrator (Fold change=1).

V-ATPase subunit F has been cloned into PCI vector and PCI-V-ATPase subunit F clones were trans formed into *E-coli* TOP 10 and therefore, DNA vaccine has been designed. Thus the effectiveness of a vaccine study requires determination of the immune response type Th1 or Th2^15^. In a previous work, it was shown that BALB/c mice vaccinated with V-ATPase subunit F have a decrease in cutaneous lesions by 41.90% in the footpad 6 weeks after parasite challenge. The decrease of footpad swelling was correlated with a 3-log reduction in parasite burden in the dLN when compared with mice immunized with the empty plasmid (Control group) (Figures 8 and 9). RT-qPCR showed vaccination of BALB\c mice with V-ATPase subunit F that could modulate the immune response, but it was insufficient for eliminating all the parasites in both inoculation site and dLNs, where the parasites survive due to existence of T regulatory cells (CD4+ CD25+) which are a major source of IL-10 and crucial for maintenance of parasite persistence in macrophage cells 19. Besides, the gene expression of IL-12 was high, but the ratio of IFN-γ to IL-4 was lower than 2 (IFN-γ\IL-4=1.6). So, the parallel fluctuations of IFN-γ, IL-12, and IL-4 transcripts are notable in the lymph node of BALB/c mice, but the parasites persist at a very high level. According to these data, it is suggested that the immune response of mice vaccinated with V-ATPase subunit F is a mixed response of Th1 and Th2, and adjuvant effect is needed to shift this response to Th1 type besides increasing IL-12 and IFN-γ gene expressions and decreasing gene expression of IL-4. This activation leads activate T cells to produce IFN-γ which in turn induces macrophages to secrete IL-12 and kill the parasites and control the infection (Figure 10).

## Conclusion

This search proved the existence of the V-ATPase subunit F gene in the genome of Syrian strain (LCED Syrian 01) *L. tropica* and in the RNA of this strain. The sequence of the cDNA of *L. tropica* V-ATPase subunit F gene was defined and submitted to the Genebank under MH124206.1 accession number. The recombinant plasmid was extracted from positive colonies and purified to obtain it as Leishmaniasis DNA vaccine. The data reported in this study suggest that there was a decrease of dermal lesion in the BALB\c mice, which were immunized with V-ATPase subunit F as DNA vaccine, but the parasite still survives at the inoculation site and in dLNs at high level. And the ratio of IFN-γ to IL-4 was 1.6. According to these data, the immune response of mice vaccinated with V-ATPase subunit F is a mixed response of Th1 and Th2 and adjuvant effect is needed to shift this response to Th1 type and improve the vaccine performance to protect against further infections that might occur in endemic areas.
